# On the Use of Ocular Douches for the Treatment of Purulent Ophthalmia of Infants, Opacities of the Cornea, &c.

**Published:** 1848-02

**Authors:** 


4. *On the Use of Ocular Douches for the treatment of Purulent Ophthalmia of Infants, Opacities of the Cornea, &c.* By M. Chassaignac.

M. Chassaignac has for the last six months employed irrigation of the eye for the treatment of the ophthalmia of young infants with the greatest success; so that while formerly blindness at the Foundling Hospital was constantly occurring from this cause, it is now seldom so produced there. The child is laid on a table, and water allowed to flow from a small tap through a tube over the surface of the eye during from 5 to 15 minutes several times a day. M. Chassaignac has discovered that in this disease a pseudo-membrane is frequently produced, the removal of which much expedites the treatment. The mortality of children suffering from disease of the eyes during the last ten years was 1 in 3; while since this plan has been adopted, it has been but 1 in 8. In the course of investigation, this means was found applicable to several other inflammatory conditions of the eye, and also especially for the removal of opacities of the cornea which resist ordinary means. Accounts of its really remarkable success in this last important application, have just been published by one of the assistants at the hospital,—*L'Union Médicale*, No. 140. *Ibid.*

